# The Impact of Sex and Performance Level on Pacing Behavior in a 24-h Ultramarathon

**DOI:** 10.3389/fspor.2019.00057

**Published:** 2019-11-06

**Authors:** Allan Inoue, Tony Meireles Santos, Florentina J. Hettinga, Daniel de Souza Alves, Bruno Ferreira Viana, Bruno de Souza Terra, Flávio Oliveira Pires

**Affiliations:** ^1^Exercise Sciences Research Laboratory, Physical Education Center Admiral Adalberto Nunes (CEFAN), Brazilian Navy, Rio de Janeiro, Brazil; ^2^Exercise Psychophysiology Research Group, School of Arts, Sciences and Humanities, University of São Paulo, São Paulo, Brazil; ^3^Exercise and Sport Sciences Postgraduate Program, Rio de Janeiro State University, Rio de Janeiro, Brazil; ^4^Operational Human Performance Postgraduate Program, Air Force University, Brazilian Air Force, Rio de Janeiro, Brazil; ^5^Physical Education Graduate Program, Federal University of Pernambuco, Recife, Brazil; ^6^Department of Sport, Exercise and Rehabilitation, Faculty of Health and Life Sciences, Northumbria University, Newcastle upon Tyne, United Kingdom; ^7^Rehabilitation Sciences Postgraduate Program, Augusto Motta University Center/UNISUAM, Rio de Janeiro, Brazil

**Keywords:** marathon, long distance running, performance, ultra-endurance, competition

## Abstract

**Purpose:** We analyzed the impact of sex, performance level and substantial speed reductions (SSR) on pacing in the VI Rio 24-h Marines Ultramarathon. This will provide insights into the importance of minimizing speed variations in relation to optimal pacing in endurance events.

**Methods:** Runners (30 males and 21 females), classified as high- (HP) and low-performance (LP) ran the race while having their time recorded every 400 m. The pacing was analyzed as the first 10% (initial epoch), the following 80% (intermediate epoch) and the last 10% of the race (final epoch). The time percentage spent at speeds <3.5 km·h^−1^ (SSR), 3.5 to 5.9 km·h^−1^ (walking speed), 6.0 to 8.0 km·h^−1^ (walk-to-running transition speed) and > 8.0 km·h^−1^ (running speed) was calculated.

**Results:** Runners showed a reverse J-shaped pacing (*P* < 0.001) regardless of sex and performance level, although male (*P* < 0.004) and HP runners (*P* < 0.001) have preserved a higher mean speed throughout the race. Male and HP runners spent more time at running speed (*P* < 0.001) and less time at SSR (*P* < 0.001) than female and LP runners. Total distance was inversely correlated with the number of SSR and speed CV in male (*r* = −0.47 and *r* = −0.64, respectively) and female (*r* = −0.61 and *r* = −0.47, respectively).

**Conclusion:** Male, HP runners showed less SSR, conserving a higher mean speed with less variation throughout the race. Results suggest that conservative pacing strategies, with lower speeds in the beginning and higher speeds toward the end, may be the most adequate for different endurance running disciplines. Results also show different competition dynamics between men and women, which warrants further exploration in ultramarathons as well as other IAAF events.

## Introduction

In endurance competitions with a known endpoint (i.e., distance or time), athletes have to constantly regulate their pace in order to finish the race in the shortest possible time or cover the largest possible distance (Abbiss and Laursen, 2008). This process is known as pacing, a key factor in optimizing endurance performance that involves the capacity to deal with physiological and perceptual responses as well as with environmental setting and race characteristics (Baden et al., 2005; St Clair Gibson et al., 2006; Esteve-Lanao et al., 2008; Tucker, 2009; Lima-Silva et al., 2010; Baron et al., 2011; Bath et al., 2012; Smits et al., 2014; Hettinga et al., 2017; Konings and Hettinga, 2018). In this regard, an inappropriate pacing strategy may result in a suboptimal performance, as athletes may have to deal with premature fatigue if, for instance, they choose an aggressive pacing strategy beyond their psychophysiological capabilities (Noakes et al., 2004, 2005; St Clair Gibson and Noakes, 2004). It is worth mentioning that, different from exercises performed at a controlled-pace such as time to exhaustion tests, self-paced exercises allow participants to pace themselves in response to physiological and perceived exertion perturbation so that a sustained pacing is maintained throughout the exercise (Tucker, 2009). This is particularly important in ultramarathons, as runners may frequently face different ground levels (i.e., sea level vs. mountains) and wind conditions in this very long running race. For example, ultramarathons held on mountains may challenge the successful pacing strategies due to climb and down phases, irregular terrains and light-to-strong winds.

In terms of endurance performances, different studies have investigated pacing strategies in running races with distances from 10 to 42 km, thus accounting for a duration ranging from ~30 min to ~2.5 h (Ely et al., 2008; March et al., 2011; Renfree and St Clair Gibson, 2013). However, running races with longer distances and durations such as ultramarathons, that have become increasingly popular in recent years, have been less investigated. This high-demanding race probably challenges the athletes' capacity to pace themselves, given the higher variation in weather conditions (Marino, 2004; Tucker, 2009), emotional responses (Baron et al., 2011), nutritional status (Jeukendrup, 2011), light and time of day (Fernandes et al., 2014; Pinheiro et al., 2016) as well as the occurrence of pain and fatigue (Millet et al., 2012). In this sense, the understanding of ultramarathon pacing and performance may be insightful to also understand other disciplines belonging to the International Association of Athletics Federations (IAAF) such as mountain running, given the challenging circumstances that athletes may face during these races. Although a few studies examining ultramarathon pacing strategies have observed a general positive pacing strategies in most parts of the event, with an increased speed in the last 10% (Lambert et al., 2004; Hoffman, 2014; Kerherve et al., 2015, 2016; Renfree et al., 2016), more studies are welcome to describe the likely beneficial pacing strategy of this race.

New insights regarding ultramarathon pacing behavior may be obtained from the analysis of the VI Rio 24-h Marines Ultramarathon dataset. For example, Bossi et al. (2017) analyzed data from 398 male and 103 female participants over five editions of this event, showing that athletes frequently use a reverse J-shaped pacing strategy in this 24-h running race. Using grouped data, the authors found that athletes used a fast start pace and then reduced the speed during the intermediate part of the race, before spurting during the final few hours. Interestingly, when using a dataset distinguished by sex, age or performance level, the authors found that neither sex nor age and performance level was related to the athletes' pacing strategy in different editions of the 24-h running race, thus indicating that a reversed J-shape pacing strategy was adopted by those athletes regardless of sex and performance level. These results contrasted previous ones in similar events (March et al., 2011; Renfree et al., 2016) which supported differences in pacing strategies between male and female runners with different performance levels. For example, while female runners are expected to be better pacers (March et al., 2011; Renfree and St Clair Gibson, 2013) likely due to differences in body size, fat metabolism and muscle fatigability (Hunter, 2016), higher performance runners usually adopt a more even pace than their slower partners (Renfree et al., 2016).

Nevertheless, more studies are required to explore the nuances of this very long race. For example, due to the prolonged duration athletes usually adopt breaks and walking periods in ultramarathons, as the completion of this race only by running seems impractical. Therefore, the use of high-frequency speed data rather than broad section averages may be needed to reveal how athletes incorporate breaks and walking periods in their pacing strategy. In fact, previous ultramarathon studies (Takayama et al., 2016; Bossi et al., 2017) have analyzed the 24-h running pacing strategy through 1 h mean data, thus likely disregarding speed variations in intervals lower than 1 h. In this perspective, it could be argued that 1 h time intervals are not long enough to accommodate important speed variations that may reflect either running or walking speeds. Moreover, another critical factor is the presence of breaks or substantial speed reductions (SSR) in ultramarathons, since it has been shown that a lower time spent with active/passive recovery periods was related to the best ultramarathon performance (Kerherve et al., 2015). Consequently, one hypothesis is that athletes planning their best 24-h ultramarathon performance may be oriented to avoid SSR. However, to the best of our knowledge no studies have attempted to describe the presence of SSR in 24-h ultramarathon races. Then, higher-frequency split data may be important to adequately describe the pacing strategy variations in this race (Angus and Waterhouse, 2011), so that analysis distinguished by sex and performance level may be more sensitive when considering the speed variations in time intervals over segments shorter than 1 h, which also allows for assessment of the presence of SSRs.

Therefore, in order to contribute to a better pacing guidance for very-long and high-demanding competitions such as a 24-h ultramarathon race, we aimed to analyze the impact of sex and performance level on the VI Rio 24-h Marines Ultramarathon pacing strategies using a higher-frequency split field data, thereby allowing us to include the number of SSR and verifying the association between SSR and 24-h ultramarathon performance. Importantly, this approach may be useful to improve the understanding of different endurance running disciplines as different studies analyzing running data from the IAAF have shown similarities in pacing profile of best male and female runners. Ultramarathon is an extreme endurance event, where any deviations from average speed are particularly pronounced, and is, therefore, a good model to explore the impact of speed variations over the race in endurance events in relation to optimal pacing.

## Materials and Methods

### Participants

This study analyzed 51 ultramarathon runners (30 males and 21 females) selected from a dataset with 140 runners of the 2013 VI Rio 24-h Marines Ultramarathon (“*VI Ultramaratona Rio 24 h Fuzileiros Navais*”). The runners selected for this study completed at least 50% of the total distance completed by the winner (214.0 km for males and 193.2 km for females). This cutoff (i.e., 50% of the distance completed by the winner) was arbitrarily defined after visual inspection of the dataset, in order to provide a sample size distinguishable by sex and performance level, but without creating inconclusive pacing strategy profiles. The selected runners were between 31 and 35 (14%), 36–40 (20%), 41–45 (14%), 46–50 (10%), 51–55 (16%), 56–60 (12%), 61–65 (4%) and 66–70 (12%) years old. The distance completed in 24 h was 166.0 ± 18.9 km and 131.5 ± 26.5 km for male and female runners, respectively. The study was approved by the Ethics Committee of the Hospital Naval Marcílio Dias (protocol 1.059.358), and waived the requirement for written informed consent for participants in this study due to raw data were already freely available in the public domain and there were no interventions, in accordance with the national legislation and the institutional requirements.

### 24-h Ultramarathon Competition

The 24-h ultramarathon was performed on a 400 m running track at the Physical Education Center Admiral Adalberto Nunes, with the running direction around the track being changed every 2 h. The winners of female and male categories were those who completed the longest distance within the 24 h. Time elapsed was recorded every lap by an electronic timing system attached to the runners' footwear. The race started in a sunny day at 09:00 a.m. on 5th October 2013, finishing at 09:00 a.m. on the following morning, maintaining a good environmental condition during all the race. Minimum and maximal temperature, as well as relative air humidity, were recorded on 5th October, between 06:00 and 12:00 a.m. (23°C, 25°C, and 77%, respectively), 12:00–18:00 p.m. (23°C, 25°C, and 57%, respectively), 18:00–00:00 p.m. (21°C, 23°C, and 64%, respectively), and on 6th October between 00:00 and 06:00 a.m. (20°C, 20°C, and 69%, respectively) and between 06:00 and 12:00 a.m. (20°C, 26°C, and 64%, respectively). The runners were allowed to consume a variety of food and beverages *ad libitum*. Time and distance records were accessed on a free website hosting the race data (http://www.chiptiempo.com/resultados/inscriptor/vi-ultramaratona-rio-24h-fuzileiros-navais-66).

### Data Analysis

We used the total distance covered within the 24 h as a performance indicator. In addition, pacing strategy analysis during this open-loop race was based on the distance completed within the 24 h. Thus, in accordance with previous works investigating the influence of the performance level on running pacing strategy (Lima-Silva et al., 2010; Bossi et al., 2017), we ranked runners according to tercile so that those runners within the lowest and highest tercile were designed as low (LP) and high performance (HP) groups, respectively. We discarded those runners within the intermediate tercile, as this ensured a comparison of pacing strategy profiles between distinguished different performance level groups. For pacing strategy analysis, we used the individual time elapsed recorded every 400 m to calculate the individual running speed within each 10 min interval, being expressed as absolute. Thereafter, based on previous literature (Bossi et al., 2017) describing a reverse J-shaped pacing strategy in ultramarathons, we analyzed the runners pacing strategy according to three different epochs (Silva et al., 2014): (1) the initial epoch, defined as the mean speed over the first 10% of the 24-h race (0 to 120 min); (2) the intermediate epoch, defined as the mean speed over the following 80% of the 24-h race (121 to 1,300 min) and; (3) the final epoch, defined as the mean speed over the last 10% of the 24-h race (1,301 to 1,440 min).

In order to accomplish the speed variations analysis, we calculated the percentage of time spent in four-speed ranges such as <3.5 km·h^−1^ (SSR), between 3.5 and 5.9 km·h^−1^ (walking speed), 6.0 and 8.0 km·h^−1^ (walk-to-running transition speed) and >8.0 km·h^−1^ (running speed). Importantly, due to the need for food, physical therapy, medical assistance, etc., athletes usually perform breaks during this challenging long-duration ultramarathon (spending time out of the track), thereby increasing the computed time and reducing the mean speed to complete a given lap. Unfortunately, the time spent at breaks during the race was unavailable in the VI Rio 24-h Marines Ultramarathon dataset, so that some estimation was required. In this regard, we determined the break periods as a substantial speed reduction (i.e., SSR) defined as a <3.5 km·h^−1^ speed, as the 3.5 to 6.0 km·h^−1^ range may represent a walking speed for most individuals (Rotstein et al., 2005). Moreover, a compendium of physical activities (Ainsworth et al., 2000) estimated an energy expenditure of 2.5 METs for ~3.5 km·h^−1^ speeds (2 mph), so that completing a 400 m lap walking at this lowest speed would suggest the presence of stop(s) rather than continuous displacement. Despite the obvious limitation of arbitrarily determining break periods, this approach allowed us to take into consideration either eventual or planned breaks. Hence, the number and duration of SSR were calculated according to this criterion, thus considering the number of occurrences with mean speed <3.5 km·h^−1^ as well as the mean and total time spent at SSR (expressed as hours), respectively.

### Statistics

We reported the results as mean ± standard deviation (s). After ensuring a Gaussian distribution, we analyzed pacing strategy through a 2 × 2 × 3 repeated-measures ANOVA, having performance level (HP vs. LP), sex (male vs. female) and epochs (initial, intermediate and final epoch) as the fixed factors, and subjects as the random factor. The Bonferroni *post-hoc* test was used in multiple comparisons, and the Greenhouse-Geisser epsilon was reported when the sphericity assumption was violated (Mauchly's test). Accordingly, a 2 × 2 × 4 repeated-measures ANOVA, having performance level (HP vs. LP), sex (male vs. female) and speed ranges (SSR, walking, walk-to-running transition and running) as the fixed factors, and subjects as the random factor, was used to analyze the speed variations throughout the race. The Bonferroni and Greenhouse-Geisser epsilon tests were further used. Additionally, a 2 × 2 ANOVA (sex vs. performance level) compared the total distance covered in the 24-h race, number and duration of each SSR (min) as well as the total time spent at SSR.

Pearson's product-moment correlation coefficients were used to determine the correlation between the number of SSR and the distance covered during the race. The coefficient of variation (CV) of the speed was determined by dividing the standard deviation by the mean speed with a sampling rate of 400 m. Moreover, the correlation between mean speed CV and total distance covered in 24 h was calculated, being reported together with the 95% confidence intervals. Based on the recommendations of Hopkins et al. (2009), values of 0.10 ≤ r < 0.30 indicate small, 0.30 ≤ r < 0.50 medium, 0.50 ≤ r < 0.70 large, 0.70 ≤ r < 0.90 very large, 0.90 ≤ r < 1.00 nearly perfect, and *r* = 1.00 perfect correlation. The significance level was set at 5% (*P* < 0.05). All analyses were performed using Statistical Package for Social Sciences (SPSS) version 21.0 (SPSS Inc., Chicago, Illinois, USA).

## Results

### Overall Pacing Strategy Responses

Overall responses identified as main effects are summarized. The 2 × 2 × 3 repeated-measures ANOVA revealed an epoch main effect (*P* < 0.001) over the 24-h ultramarathon so that, regardless of sex or performance level, runners showed a faster start pace as the speed in the initial epoch was higher than speed in the second and third epochs (*P* < 0.001), but no differences were observed between the second and third epochs (*P* = 0.398). Thus, the overall pacing profile was a reverse J-shaped pacing strategy characterized by a fast start pace in the initial 10% of the race (initial epoch, 9.5 ± 1.5 km·h^−1^), followed by a progressive decline in mean speed during the following 80% of the race (intermediate epoch, 6.2 ± 1.4 km·h^−1^), before a non-significant endspurt in the last 10% of the race (final epoch, 6.5 ± 1.4 km·h^−1^). Moreover, male runners ran the ultramarathon (*P* = 0.000) faster (7.92 ± 1.79 km·h^−1^) than female runners (6.66 ± 2.18 km·h^−1^). Accordingly, HP runners (8.25 ± 1.90 km·h^−1^) were faster (*P* < 0.001) than LP runners (6.55 ± 1.83 km·h^−1^).

### Sex by Performance Level Interaction Effects on Pacing Strategy

**Figure 1:**
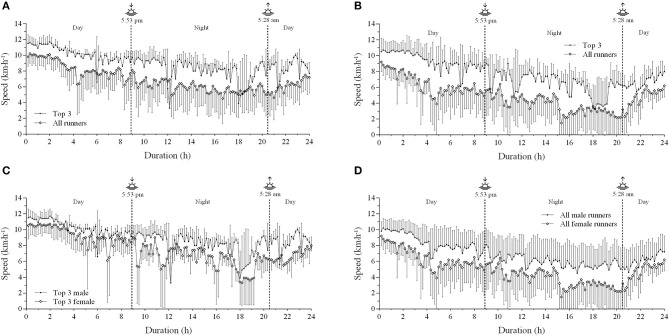
Mean running speed in each 10-min interval of the 24-h ultramarathon. **(A)** top 3 male and all male runners; **(B)** top 3 female and all female runners; **(C)** top 3 male and top 3 female runners and **(D)** all male and all female runners; Top 3, the overall 3 best runners.

**Figure 2:**
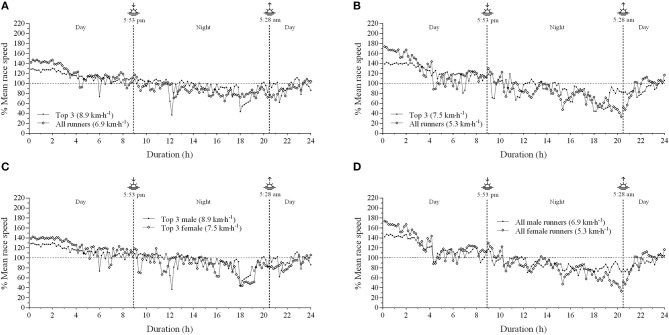
Relative mean race speed during the 24-h ultramarathon. **(A)** top 3 male and all male runners; **(B)** top 3 female and all female runners; **(C)** top 3 male and top 3 female runners **(D)** all male and all female runners; Top 3, the overall 3 best runners. Mean speed was reported in brackets.

Multiple comparisons revealed an interaction effect between performance level and epochs. For example, LP male runners started the race at a higher relative mean speed (144.2 ± 16.3%) when compared to HP male runners (133.2 ± 13.1%). Accordingly, LP female runners started at a higher relative mean speed (198.8 ± 24.3%) than HP female runners (149.6 ± 22.3%). Furthermore, a sex by performance level interaction effect was observed in pacing strategy. Interestingly, no difference was observed in absolute mean speed between HP male and HP female runners in the initial epoch (*P* < 0.165). However, HP male runners ran faster than female ones in intermediate (*P* < 0.019) and final epochs (*P* < 0.003). In contrast, LP male runners were faster than LP female runners in initial (*P* < 0.012), intermediate (*P* < 0.000) and final epochs (*P* < 0.004). Figure 1 and Figure 2 depict the pacing strategy profile of the overall 3 best male and female runners and all runners.

**Figure 3:**
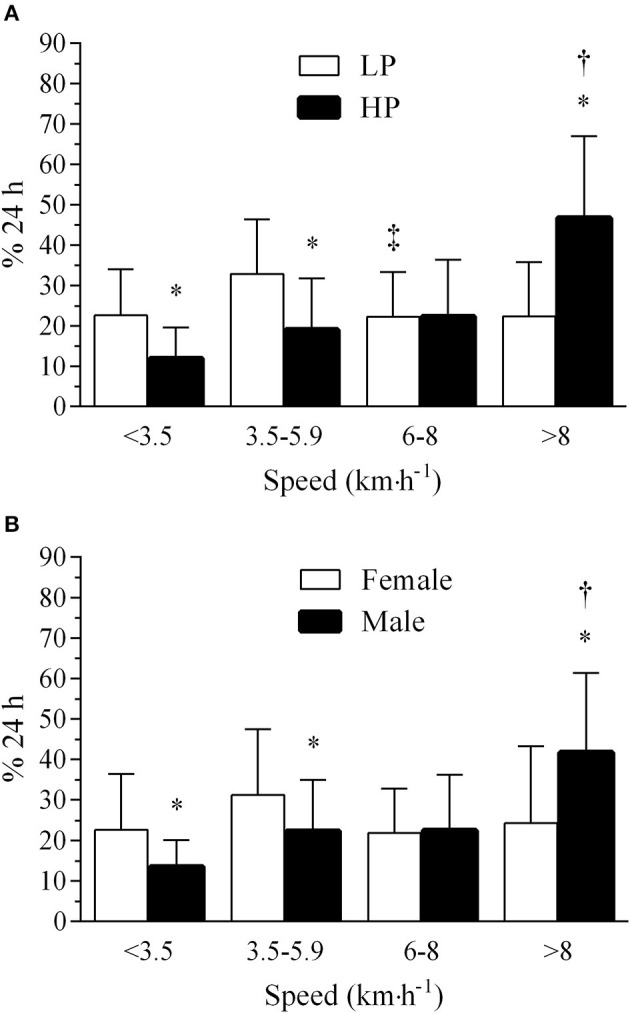
Percentage of time spent at speeds corresponding to a substantial speed reduction (SSR <3.5 km·h^−1^), walking (from 3.5 to 5.9 km·h^−1^), walk-to-running transition (from 6.0 to 8.0 km·h^−1^) and running (>8.0 km·h^−1^). **(A)** HP vs. LP; **(B)** male vs. female; *, significant difference (*P* < 0.05) between performance levels or sexes in the same speed range; ‡, significant difference (*P* < 0.05) when compared to 3.5–5.9 km·h^−1^ LP; †, significant difference (*P* < 0.05) when compared to lower speed range(s) in the same performance level or sex; LP = low performance; HP, high performance. Results presented in mean and standard deviation.

Regarding the time spent in different speeds, the 4 × 2 × 2 repeated-measures ANOVA showed a speed by sex interaction effect (*P* < 0.004) as well as a speed by performance level interaction effect (*P* < 0.001). Thus, overall results were that male and HP runners spent more time in running speeds (>8.0 km·h^−1^) than female and LP runners. Accordingly, male and HP runners spent less time at SSR (i.e., speed <3.5 km·h^−1^) and walking speed (i.e., 3.5–5.9 km·h^−1^) than females and LP, respectively. Figure 3 shows the relative time spent in different speed ranges during the 24-h ultramarathon race according to performance level (panel A) and sex (panel B).

### Substantial Speed Reductions Analysis

When the time spent in SSR was analyzed, a significant performance level by sex interaction effect in the number (*P* = 0.037) and total time of SSR (*P* < 0.001) was observed, but not for the mean duration of each SSR (*P* = 0.067). Overall results were that LP female runners showed an increased number of SSR when compared to HP female runners (*P* < 0.001), thereby spending a higher total time in SSR than HP female (*P* < 0.001). Furthermore, LP female runners spent a higher total time in SSR than LP male runners (*P* < 0.001). As a result, there was a significant performance level by sex interaction effect in total distance covered during the 24-h race (*P* < 0.001), as male runners ran longer distances than their female partners in HP and LP groups (Table 1).

**Table 1:** Total distance, number of substantial speed reductions (<3.5 km·h^−1^), mean duration of each substantial speed reduction, total time in substantial speed reduction, and %total time (24 h) in substantial speed reduction of performance level groups and sex.

**Variables**	**Performance level**	
	**HP**	**LP**
Male	(*n* = 10)	(*n* = 10)
Total distance (km)	186.7 ± 18.2	149.5 ± 3.1^a^
Number of SSR	11 ± 4	13 ± 4
Mean duration of each SSR (min)	16.9 ± 28.6	16.0 ± 23.4
Total time in SSR (h)	3.0 ± 1.7	3.5 ± 1.5
%Total time (24 h) in SSR	12.5 ± 7.1	14.6 ± 6.3
Female	(*n* = 7)	(*n* = 7)
Total distance (km)	162.2 ± 19.0^b^	106.1 ± 3.7^a^^,^^b^
Number of SSR	6 ± 3	15 ± 5^a^
Mean duration of each SSR (min)	25.5 ± 44.8	32.1 ± 17.5
Total time in SSR (h)	2.7 ± 2.2	8.1 ± 1.5^a^^,^^b^
%Total time (24 h) in SSR	11.3 ± 9.2	33.8 ± 6.3^a^^,^^b^

*HP, High performance; LP, Low performance; SSR, Substantial speed reduction*.

a *significant difference (P <0.001) between performance levels in the same sex*.

b *significant difference (P <0.001) between sexes in the same performance level. Results presented in mean and standard deviation*.

**Figure 4:**
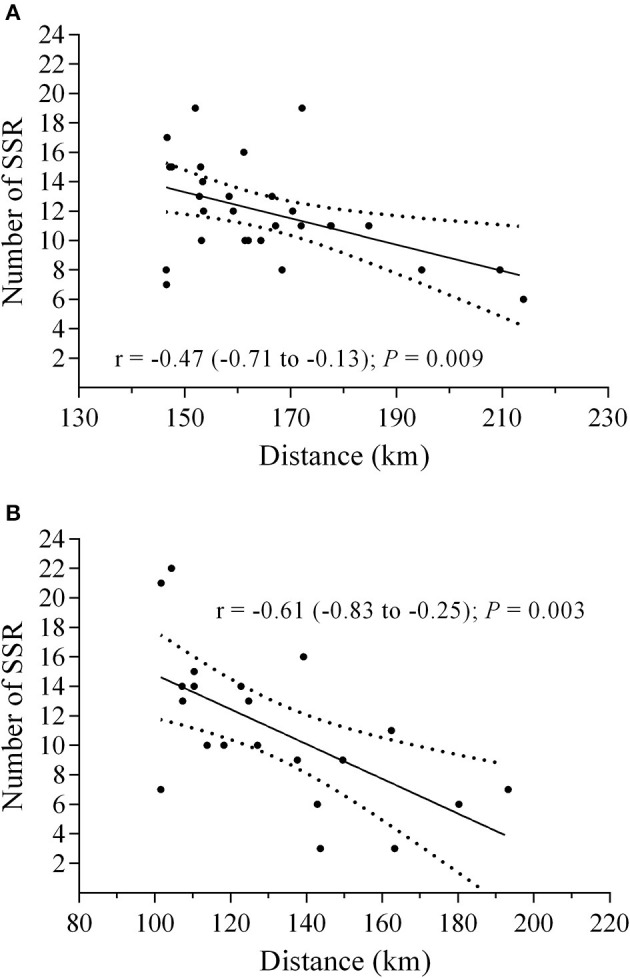
Correlation between number of substantial speed reductions (SSR) and total distance. **(A)**, male (*n* = 30); **(B)**, female (*n* = 21).

**Figure 5:**
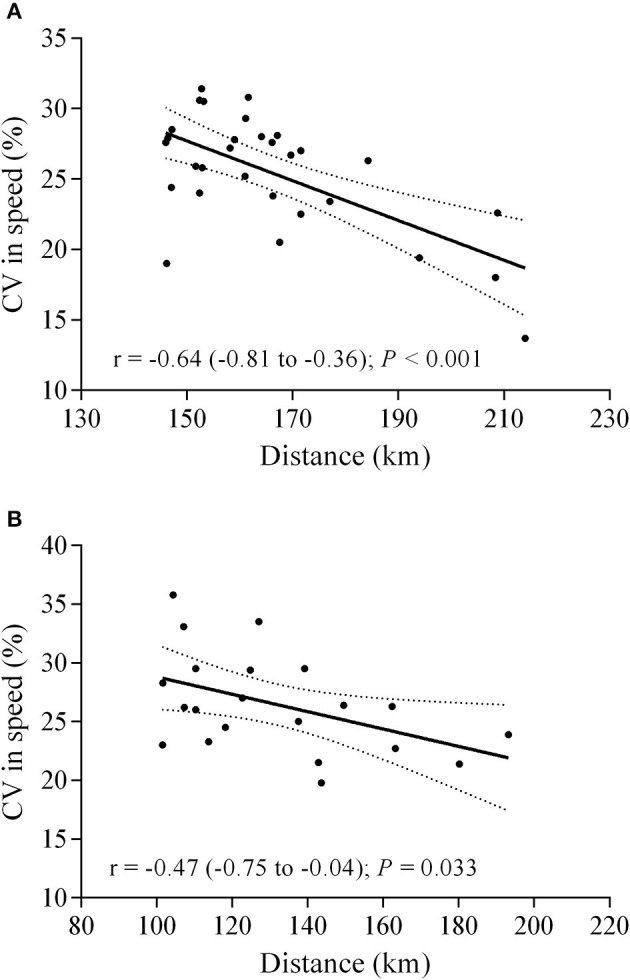
Correlation between speed coefficient of variation (CV) and total distance. **(A)** male (*n* = 30); **(B)** female (*n* = 21).

Significant negative correlations were observed between the number of SSR and the total distance covered in 24 h in both male (*r* = −0.47; *P* = 0.009) and female runners (*r* = −0.61; *P* = 0.003) (Figure 4). Accordingly, there was a significant negative correlation between speed CV and total distance covered in 24 h, in both male (*r* = −0.64; *P* < 0.001) and female runners (*r* = −0.47; *P* = 0.033). The lowest speed variation was found in HP groups, both male (21.5 ± 4.8%; 17.1 to 25.9%) and female runners (23.1 ± 2.5%; 20.8 to 25.5%). In contrast, LP male (27.2 ± 3.0%; 24.5 to 30.0%) and female runners (28.8 ± 4.4%; 24.8 to 32.9%) showed the higher levels of CV in speed (Figures 5A,B).

## Discussion

The novel main finding of the present study suggests that the number of breaks may partially explain the 24-h ultramarathon performance as HP runners spent less time in speeds <3.5 km·h^−1^ (SSR) than LP runners. Accordingly, male runners, regardless of performance level, also showed a lower time (%24 h) in SSR than female runners. Together, both results suggest a lower time spent at very low speeds associated with active or passive resting (walking speed from 3.5 to 5.9 km·h^−1^ or SSR <3.5 km·h^−1^) is related to better 24-h ultramarathon performance. Importantly, HP runners showed a more even pace with less speed variations than LP ones, perhaps indicating that a more conservative pacing strategy is the most appropriate to attain optimal performance in endurance events performed in extreme conditions.

In contrast to previous ultramarathon studies (Kerherve et al., 2015, 2016; Renfree et al., 2016), but confirming others (Bossi et al., 2017), the present study showed that runners adopted a reverse J-shaped pacing strategy during the VI Rio 24-h Marines Ultramarathon running, regardless of sex and performance level. Thus, runners slowed down the pace during most of the race, after performing a faster start and before spurting at the last 10% of the race. In this regard, HP runners sustained a higher mean speed and lower variation throughout the 24-h race than LP runners, regardless of sex differences. Importantly, both male and female HP runners used a more conservative pacing strategy, as they ran the first ~3 h at a lower relative speed (133.2 and 149.6% of the mean speed, respectively) than LP ones (144.2 and 198.8% of the mean speed, respectively). Similar results have been found by Renfree and St Clair Gibson (2013) during the women's world championship marathon race, as they observed that underperformance was likely related to a less conservative pacing strategy, characterized by initial speeds that were unsustainable for the entire distance. Additionally, finishing times of the best athletes were closer to their personal best time performance, being the averaged speed at 98.5 ± 1.8% of the speed achieved in their personal best time performance. In contrast, athletes from the other groups showed a reduced averaged speed (at 92.4 ± 4.4%) when compared to the best runners. In our ultramarathon dataset, this more conservative pacing strategy possibly allowed racers to perform a lower number of breaks and speed variations throughout the race (as argued in the next section). Thus, one may argue that all these findings, together, suggest that conservative pacing strategies may be more adequate for different endurance running disciplines.

### Variation of Speed and the Presence of Breaks

A remarkable aspect of ultramarathon races is the high-speed variation (Parise and Hoffman, 2011; Hoffman, 2014). Accordingly, we found a high-speed variation during the VI Rio 24-h Marines Ultramarathon (Figure 1), which was higher than that reported by previous studies. For example, a study investigating the pacing strategy in 24 editions of a mountain 161 km ultramarathon (*Western States Endurance Run*) observed that winners showed a speed CV about 12%, while the ten best finalists were between 9 and 13% (Hoffman, 2014). Ely et al. (2008) reported that elite runners showed very little changes in 5-km pace segments during a marathon race, thus suggesting a low pace variability. In addition, Santos-Lozano et al. (2014) showed a lower speed variability in top runners, given the 5-km splits CV ranging 6.6 to 7.8 and 8.3 to 14.4% in more and less successful runners, respectively. In this regard, an even pace profile avoiding an excessively fast start pacing strategy may be important to avoid premature fatigue as the race progresses and a decrease in speed in the second half of the race. Our CV results are similar to those of Takayama et al. (2016), as they reported that CV of speed was moderately correlated with total distance covered (*r* = −0.68; *P* < 0.001).

In the present study, we observed a speed CV ~21% for male and ~23% for female HP runners. The presence of breaks, arbitrarily defined as a mean speed <3.5 km·h^−1^ (i.e., SSR), may be related to this higher speed CV, as athletes use the SSR sections to recovery or feed themselves, as well as to take part in physiotherapy and medical assistance, etc., during a 24-h ultramarathon running. Anecdotal information indicated that most participants of the VI Rio 24-h Marines Ultramarathon believed that breaks were beneficial for performance so that they included breaks in their strategy to complete the race. However, previous literature has shown conflicting results, as Kerherve et al. (2015) reported that the lower stop total time, the better the performance in a 106 km mountain ultramarathon (*r* = −0.772; *P* = 0.001; 95% confidence interval = −1.15 to 0.39) while Kerherve et al. (2016) did not find a significant relationship (*r* = −0.35; *P* = 0.21) between stop total time and performance in a hilly terrain ultramarathon. In the preset study, we used a high-frequency split dataset and found a significant inverse correlation between the number of SSR and the total distance covered in 24 h. Taking together, previous literature and the present results may indicate that, at least to a flat terrain 24-h ultramarathon, best performance may be related to a lower number of breaks (i.e., SSR), suggesting that the maintenance of running speeds with less speed variations is beneficial to this long race. Future studies are welcome to confirm this suggestion in other ultramarathons and verify the influence of the different terrains.

Interestingly, observations of overnight vs. daytime pacing profile further suggest a possible change in pacing across the lighting-dark transition phases in the 24 h, not only in the top 3 runners but in the other runners as well, being more pronounced in the female runners. Accordingly, the SSR number was apparently higher overnight in the LP female runners. However, it is difficult to know if such an apparent nighttime-daytime pacing profile difference was due to circadian cycle variations or accumulated competition hours, as the altered pacing observed overnight may have reflected the accumulated number of hours competing. Actually, there was a more pronounced change at 9 p.m. and 3–4 a.m., thus representing an accumulation of 12 and 18–19 h of competition from the start line, respectively. Future studies are required to address to this interesting issue.

### Influence of Performance Level on Pacing Strategy

Our data corroborate a recent study analyzing the influence of the performance level in pacing strategy during a 100 km ultramarathon (Renfree et al., 2016). The best performance group raced the first 30 km at lower relative speeds when compared to other groups in the first three 10-km segments (all *P* < 0.01). In addition, our results corroborate with findings by Bossi et al. (2017), who showed that the fastest runners showed a more conservative initial speed (initial 3 h of the race), before slowing down as the competition progressed. In contrast, slower runners were unable to maintain their initial speed as much long as the fastest runners, thereby reducing their mean speed in a higher extension. Similar results were reported for elite marathon runners during the women's World Championships marathon in 2009 (Renfree and St Clair Gibson, 2013), as runners finishing the race in the first quartile raced the first two 5 km segments at a slower relative speed when compared to those finishing the race between the second and the fourth quartile. Esteve-Lanao et al. (2014) described the pacing distribution of 768 male runners participating from 2007 to 2013 of the world cross-country championships. Groups of 10 participants according to final position (1st to 10th, 11th to 20th, etc.) were considered. They reported that top-10 finishers in the world cross-country championships elicited an even pace rather than other finishers that used a fast-to-slow pacing strategy pattern, consequently, a much more stable pacing pattern should be considered to maximize the final position. With the purpose to examine pacing among 48 male runners who ran more than 161 km in a 24-h ultramarathon, Takayama et al. (2016) divided runners into five groups (A: 1st−10th, B: 11th−20th, C: 21th−30th, D: 31th−40th, and E: 40th−48th). The 24-h distance within the various groups ranged from 238.38 ± 11.41 km for group A to 164.14 ± 2.49 km for group E. Group A runners ran at a relatively constant speed (>8 km·h^−1^) during the second half of the race, whereas the corresponding pace was slower (<6 km·h^−1^) for other groups.

In the present study, we observed a slower relative speed at the initial ~3 h of the race in HP than in LP runners, regardless of sex. Possibly, this more aggressive pacing strategy may have been related to a decreased performance in LP runners, as the maintenance of a greater initial relative speed along the race may have been unsustainable. Thus, the selection of a more aggressive start pacing strategy may have led LP runner to reduce the mean speed and possibly perform more SSR. Consequently, these results provide important considerations for coaches and athletes competing in endurance events, as they may suggest that an aggressive start pacing strategy as performed by LP runners is possibly inadequate. For athletes competing in different IAAF running disciplines, one may suggest that the adopted pacing strategy has a major influence on the final achievement, and a more conservative pattern is advised. Furthermore, this result is different from that reported by Lima-Silva et al. (2010) during a simulated 10 km running race, as the LP runners investigated in that study started the running race with a more conservative pacing strategy when compared to HP runners. However, it is worth to suggest caution when comparing both studies, given the difference in distance and duration between a 24-h ultramarathon running and a 10 km running race (Lima-Silva et al., 2010). For example, a 10 km running race is as long as only ~3% of a 24-h ultramarathon race, thus suggesting long-term races represent a different psychophysiological challenge for the athlete.

### Differences of Sex in Pacing Strategy

Recently, Bossi et al. (2017) showed that athletes adopted a reverse J-shaped pacing strategy in a 24-h ultramarathon, with low deviations from the mean speed during most of the race and the presence of an endspurt in the last hours (despite slight reductions). Accordingly, we found an increased speed in LP and HP groups in the last hour, however, while female runners increased the speed throughout the last 10% of the race, male runners showed a variable endspurt according to the performance level (LP male runners spurted throughout the last 10% of the race and HP runners slowed down the pace in the last 30 min of the endspurt phase). The reason for this discrepancy between studies is not clear (Bossi et al., 2017). Perhaps the higher data sampling frequency used in the present study may have allowed us to identify this difference in pacing strategy.

A study by Renfree et al. (2016) reported that women demonstrated lower initial relative speeds when compared to men. It is possible that the decision for a higher initial speed in female runners in the present study has been influenced by the speed imposed by male runners since both men and women started the ultramarathon race at the same time. This suggestion is based on the “herd principle,” that the most likely decision that an athlete could make about the selection of an initial pace during a competitive event is simply to follow the behavior of direct competitors, as shown in 4 km time trials raced against opponents (Konings et al., 2016). Likewise, such a “herd principle” may be present in findings reported by Renfree and St Clair Gibson (2013) in women World Championship marathon race as well as Hanley (2014) and Esteve-Lanao et al. (2014) in IAAF World Cross Country Championships.

## Limitations

Two obvious limitations of the present study were related to the SSR calculation. First, the absence of information related to planned or eventual SSR may limit our interpretations, as we were unable to correlate some behaviors normally linked to breaks in a pacing strategy perspective (such as planned meal, physiotherapy, etc.). Second, the use of an arbitrary criterion to identify periods of break may be also considered as a limitation, as some reductions in mean speed may have been inadequately identified as a break; in contrast, they may indicate a substantial speed reduction without a complete break. However, most behaviors such as feeding may be still accomplished at a very slow walk, so that the SSR strategy used in the present study may be an indication of how SSR may impact 24-h ultramarathon pacing strategies. The choice of speed threshold was based on previous papers of human locomotion (Yokoyama et al., 2016; Fokkema et al., 2017). The selection of slow speed walk of 4 km·h^−1^ as reported elsewhere (Yokoyama et al., 2016), was important to make the identification of a SSR possible. Another limitation is the absence of knowledge regarding the experience and training level of the runners.

### Practical Applications

The findings of the present study may be used by coaches and athletes to plan and develop more effective pacing strategies for ultramarathon races. For example, they indicate that starting an extreme endurance race at a lower percentage of the mean speed, thus decreasing the difference between the initial and the mean speed that could be sustained throughout the race may be beneficial for long-term race performance. Mainly in ultramarathon races, a more conservative pace maintaining a mean speed with lower variation allows the avoidance of a number of substantial speed reduction during the race (i.e., SSR, speeds <3.5 km·h^−1^) and increasing the time in running speeds >8.0 km·h^−1^), may improve performance in 24-h ultramarathons. Therefore, feeding, clinical assistance, and other behaviors could be planned in a pacing strategy scenario that considers this information.

The results of the present study may be applicable to ultramarathon runners having a similar performance level. We worked on a public dataset of 140 runners competing the VI Rio 24-h Marines Ultramarathon race and unfortunately, no data to characterize them was available. Study by Knechtle et al. (2011) reported anthropometric and training experience of recreational ultramarathon runners completing 146.1 ± 43.1 km in a 24-h ultramarathon in Basel, Switzerland (2008 to 2010). In contrast, Takayama et al. (2016) analyzed the best 48 male runners who ran more than 161 km in a 24-h ultramarathon in Tokyo, Japan (2014), as a contest to select members of Japan's national team for the world championships. Runners completed from 258.7 to 164.1 km in this later ultramarathon. In the present study, most of the male runners completed from 148 to 192 km while most female ones completed from 100 to 160 km. Consequently, one may argue that our pacing strategy results are directly applicable to recreational runners, similar to those investigated by Knechtle et al. (2011). Future studies may confirm these results in ultramarathon runners having a performance level similar to those athletes investigated by Takayama et al. (2016).

Results of the present study may also provide insights into the 2019 IAAF World Championship in Qatar regarding a physiological and psychological highly-demanding, given the likely hot temperatures and potentially high humidity levels found in September and October that may eventually impair performance in endurance events. Despite this 2019 Championship will not run Ultramarathon and Mountain Running races in its timeline, high temperatures and humidity levels may probably push the human body limits in Marathon and 50 km Race Walk competitions toward those usually seen in ultra-long running. For example, the reversed-J pacing strategy observed in the present ultramarathon dataset may likely indicate that athletes competing in these races.

## Conclusion

Regardless of sex and performance level, runners used a reverse J-shaped pacing strategy during a 24-h ultramarathon race. Importantly, male and female HP runners showed that a conservative pacing strategy, with lower speeds in the beginning and higher speeds toward the end (avoiding substantial speed reductions such as SSR), may be the most adequate for different endurance running disciplines. These results also show that different competition dynamics between men and women warrant further exploration, given the possibility of a “herd principle.”

## Data Availability Statement

The datasets generated for this study are available on request to the corresponding author.

## Ethics Statement

The studies involving human participants were reviewed and approved by Ethics Committee of the Hospital Naval Marcílio Dias. Written informed consent for participation was not required for this study in accordance with the national legislation and the institutional requirements.

## Author Contributions

AI, TS, FH, DA, BV, BT, and FP have made a substantial, direct and intellectual contribution to the work, and approved it for publication.

### Conflict of Interest

The authors declare that the research was conducted in the absence of any commercial or financial relationships that could be construed as a potential conflict of interest.
